# Trained Immunity in Health and Disease

**DOI:** 10.1002/mco2.70461

**Published:** 2025-11-06

**Authors:** Meng Yao, Jian Zhou, Jialun Mei, Chuan Gao, Peng Ding, Gan Li, Changqing Zhang, Zhiwei Li, Junjie Gao

**Affiliations:** ^1^ Department of Orthopaedics Shanghai Sixth People's Hospital Affiliated to Shanghai Jiao Tong University School of Medicine Shanghai China; ^2^ Institute of Microsurgery on Extremities Shanghai Sixth People's Hospital Affiliated to Shanghai Jiao Tong University School of Medicine Shanghai China; ^3^ Division of Hepatobiliary and Pancreatic Surgery Department of Surgery The First Affiliated Hospital Zhejiang University School of Medicine Hangzhou Zhejiang China

**Keywords:** epigenetic inheritance, immunotherapy, immune cells, metabolic reprogramming, trained immunity

## Abstract

Trained immunity as a critical regulator of host defense and disease pathogenesis bridges the gap between innate and adaptive immunity. For decades, the classic dichotomy of innate immunity and adaptive immunity has shaped our knowledge of immune function. Innate immunity has traditionally been regarded as a rapid, nonspecific first line of defense without memory capacity, while adaptive immunity is characterized by slower, antigen‐specific responses and long‐term immune memory. However, emerging evidence that innate immunity exhibits memory‐like properties challenges the paradigm. Basically, innate immune cells with nonspecific memory retain functional imprints of prior encounters with diverse stimuli. Here, we comprehensively explore the intricate molecular and cellular mechanisms that underpin trained immunity, encompassing epigenetic inheritance, metabolic reprogramming, and transcriptional rewiring. Its dual roles are highlighted in health and disease. On one hand, it bolsters host defense against a broad spectrum of pathogens from bacteria to viruses, and enhances vaccine efficacy through heterologous protection. On the other hand, its dysregulation contributes to infection, inflammation, and cancer progression. As for the promising opportunities on therapeutic intervention, the challenges in precisely modulating trained immunity are tackled to offer a holistic perspective on the dynamically evolving field.

## Graphical abstract

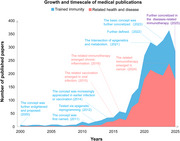

## 1 Introduction

Innovative therapeutic approaches to stimulate or inhibit trained immunity broaden the potential implications in health and disease [1]. As prevailing notion that only adaptive immunity could exhibit memory was challenged by emerging evidence [2, 3, 4]. Trained immunity was divided into two distinct phases, innate immunity (rapid, nonspecific, and “amnesic”) and adaptive immunity (slower, antigen‐specific, and capable of generating durable memory) [5]. It involves immune cells against infection, inflammation, and cancer in terms of epigenetic inheritance, metabolic reprogramming, and transcriptional regulation. The current knowledge enables the mechanisms underlying cell‐type‐specific differences in trained immunity further clarified to facilitate the clinical application of trained immunotherapy.

**FIGURE 1:**
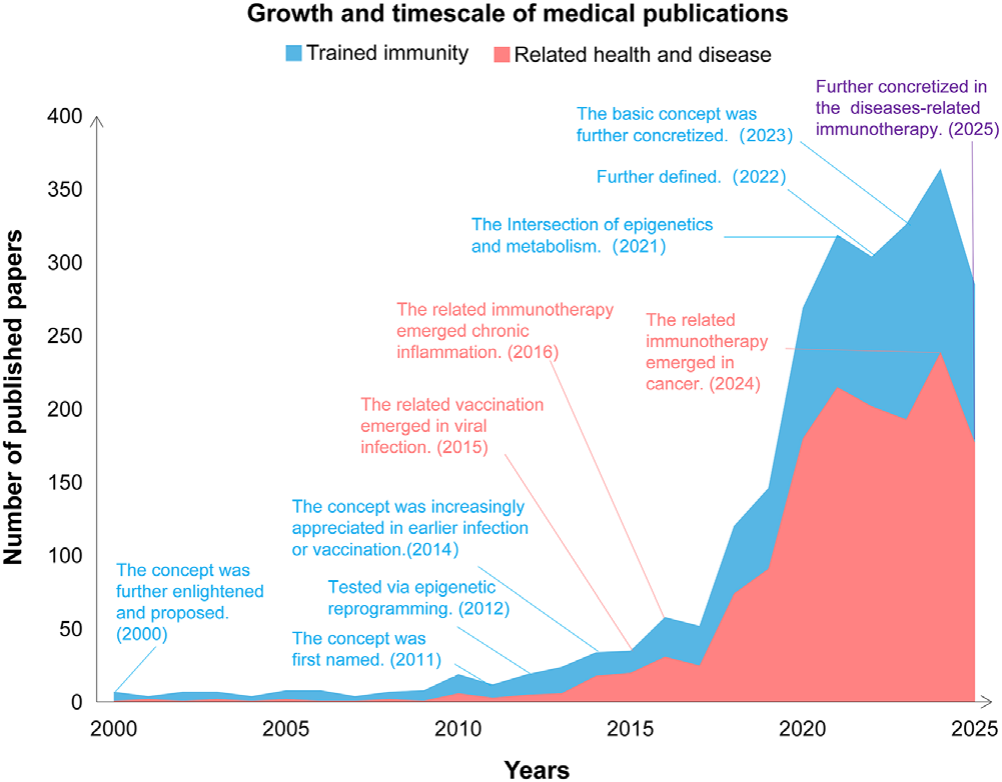
Growth and timescale of medical publications on trained immunity and its related health and disease. This figure illustrates the number of published papers over time, from 2000 to 2025 (by August 14, 2025), related to trained immunity and its related health and disease. The data are visualized through two layered area graphs, each representing a different category of publications: research on trained immunity (blue area), its related diseases (red area), and purple represents the combination of the two entities. This graph underscores the expanding research and clinical importance of trained immunity and its related diseases. The *x*‐axis represents the ages, ranging from 2000 to 2025, and the *y*‐axis quantifies the number of published papers. After trained immunity was named in 2011 to describe the memorization of innate immunity, the literature in related fields increased. We mapped the development of trained immunity and its related health and disease, and marked the nodes where key events occurred over the past 25 years.

Since trained immunity was coined by Netea et al. [6] to describe the functional reprogramming of innate immune cells after initial stimulation, resulting in enhanced secondary responses, relevant researches have expanded exponentially. In detail, the number of publications on both trained immunity and related health and disease has increased significantly after 2011, indicating that these fields have received extensive attention and in‐depth development in recent 25 years (Figure 1). This graph marks significant milestones from 2000 to 2025 to underscore the expanding research and clinical importance of trained immunity and its related health and disease. Therein, mechanistic insights that epigenetic and metabolic reprogramming were identified as core drivers [7, 8, 9, 10, 11]. The phenomenon extended beyond traditional innate cells to nonimmune cells, broadening its relevance [12, 13]. In recent years, trained immunity has implicated in health and disease like infection, inflammation, and cancer [14, 15, 16, 17, 18]. These discoveries highlight its dual role as a protective mechanism and a pathogenic force. Despite this progress, critical gaps remain. The cell‐type‐specific differences in trained immunity need to be further characterized, and the molecular crosstalk between metabolic and epigenetic pathways is not fully elucidated [19, 20]. Moreover, while trained immunity holds promise as a therapeutic target, its long‐term safety and clinical translatability require further investigation [21]. The above issues have hindered the connection between basic research and clinical application.

In order to bridge basic science and clinical application, the review aims at clarifying these issues by synthesizing the history, mechanisms, and disease relevance of trained immunity. First, the historical milestones and current research status in trained immunity are delineated. Trained immunity across cell types and core mechanisms are compared, followed by disease‐specific implications. Finally, we critically evaluate therapeutic strategies ensuring logical continuity from basic principles to clinical applications.

## 2 Cells Involved in Trained Immunity

Unlike adaptive immunity that relies on T/B cell clonal expansion, trained immunity is mediated by innate immune cells that undergo persistent molecular and functional reprogramming, including epigenetic inheritance and metabolic reprogramming [22, 23]. Innate immune cells relying on epigenetic inheritance and metabolic reprogramming in persistent trained state are enabled to maintain enhanced function independently of antigen specificity. The changes “prime” cells (Table 1) to mount more robust defenses upon re‐exposure to threats.

**TABLE 1:** Cell types with trained immunity.

Cell	Stimulant	Function	References
Alveolar macrophage	Lipolyaccharide	Increased responsiveness to pathogens	[24, 25]
Neutrophil	β‐Glucan, Bacille Calmette–Guérin (BCG)	Inhibition of tumor growth, enhanced antibacterial function	[26, 27]
Monocyte	Catecholamines, β‐glucan, BCG	Persistent proinflammatory induce or maintain autoimmune disease	[28, 29, 30]
Natural killer cell	BCG	Improves responsiveness to unrelated microbial stimuli	[31, 32]
Macrophage	Candida albicans	Promote innate antiviral immunity	[18, 30]
Kupffer cell	Preoperative exercise	Protects the organ from inflammatory damage	[33]
HSC	Lipolyaccharide, *M. tuberculosis*	Initiating a secondary immune response more effectively, impairs trained immunity, causing immune escape from *Mycobacterium tuberculosis*	[34, 35]
Skin epithelial stem cell	Model of skin inflammation induced by TLR7 and NALP3 agonist imiquimod	Promotes tissue healing	[36, 37]
Urothelial cell	UPEC	Affecting susceptibility to recurrent UTI	[38]

Abbreviations: BCG: Bacillus Calmette–Guérin; HSC: Hematopoietic stem cells; NALP3: Neutrophilic alkaline phosphatase 3; TLR7: Toll‐like receptor 7; UPEC: Uropathogenic Escherichia coli; UTI: Urinary tract infection.

### 2.1 Monocytes and Macrophages

Circulating monocytes in the bloodstream are rapidly recruited to sites of infection, inflammation, or cancer, where they differentiate into macrophages [28, 30]. Macrophages, central effector cells in trained immunity, can be trained by diverse stimuli, including microbial components such as fungal β‐glucans or bacterial lipopolysaccharides (LPS) [39]. Trained macrophages display enhanced phagocytic activity, increased production of reactive oxygen species (ROS) and nitric oxide (NO), and elevated secretion of proinflammatory cytokines. Additionally, their capacity to recognize and respond to pathogens is modulated by changes in surface receptor expression. For instance, the upregulated pattern recognition receptors (PRRs), notably Toll‐like receptors (TLRs), leads to heightened sensitivity to pathogen‐associated molecular patterns [40]. It highlights the central role of the monocyte–macrophage axis in trained immunity to provide critical therapeutic targets for managing diseases such as infection, inflammation, and cancer.

### 2.2 Natural Killer Cells

Natural killer (NK) cells, pivotal for antiviral and antitumor immunity, exert critical functions in the early defense against pathogens and transformed cells [31, 41]. NK cells undergo functional reprogramming upon stimulation with proinflammatory cytokines. Enhanced NK cell effector function is trained by cytotoxicity, interferon‐γ (IFN‐γ) secretion, and altered receptor profile. Notably, a key distinction from adaptive immunity that trained NK cells mount more rapid and robust responses to secondary challenges independent of prior antigen‐specific activation due to clonal expansion of antigen‐specific T/B cells [23]. These findings highlight NK cells as critical contributors to trained immunity, bridging innate and adaptive immune responses. Dissecting the reprogramming pathways holds promise for developing novel immunotherapies targeting viral reactivation, chronic inflammation, and cancer.

### 2.3 Innate Lymphoid Cells

Pivotal functions in early immune defense, tissue homeostasis, and the regulation of inflammation are exerted by innate lymphoid cells (ILCs), a heterogeneous family of innate lymphocytes devoid of antigen‐specific receptors [42]. Trained immunity modulates distinct ILC subsets with subset‐specific functional outcomes, extending the role of these cells beyond acute responses to long‐term immune regulation. ILC2s, critical mediators of type 2 immunity, undergo training in response to cytokines and alarmins [43]. Augmented secretion of type 2 cytokines caused by the reprogramming are key drivers of eosinophil recruitment, mucus production, and helminth clearance. Notably, trained ILC2s retain enhanced effector function for weeks, enabling faster and more robust responses to secondary challenges. The changes have dual implications that they support protective immunity against helminths but may also exacerbate allergic pathologies by perpetuating type 2 inflammation. ILC3s subject to trained immunity are essential for defending against extracellular bacteria and fungi. Their defense mediated by epigenetic inheritance and metabolic reprogramming enhances production of proinflammatory cytokines and strengthens mucosal barrier function. Trained ILC3s improve intestinal epithelial repair and limit pathogen dissemination, which makes them potential targets for treating inflammatory or respiratory infections [44]. Like other innate immune cells, ILCs rely on epigenetic inheritance and metabolic reprogramming to “lock in” the trained state. The persistent changes enable ILCs to maintain enhanced function independently of antigen specificity.

Collectively, current findings position ILCs as key regulators of innate immune memory, bridging early inflammation to long‐term tissue and systemic immunity. Their subset‐specific responses to training highlight the complexity of trained immunity and its role in health and disease. Dissecting the molecular mechanisms underlying ILCs training may unlock novel therapeutic strategies for conditions ranging from allergic diseases to mucosal infections.

### 2.4 Other Cells

Dendritic cells (DCs), epithelial cells, and hematopoietic stem cells (HSCs) or progenitor cells can all undergo trained immunity [45, 46, 47]. After being exposed to training stimuli, antigen‐presenting ability of DCs is enhanced, the expression of costimulatory molecules is increased, and the secretion of cytokines is altered, resulting in T cells being more efficiently activated to enhance adaptive immunity. When epithelial cells are stimulated by pathogens or inflammatory mediators, epigenetic and transcriptional changes are induced, leading to the continuous upregulation of antimicrobial peptides, proinflammatory cytokines, and chemokines, thereby enhancing local defense. Additionally, when HSCs are stimulated by systemic inflammation or training stimuli, their differentiation potential is altered by epigenetic inheritance, generating functionally enhanced myeloid and lymphoid progeny. The process termed “central trained immunity” mediates long‐term immune regulation [48]. Notably, these cell‐specific adaptations underscore the integrated, memory‐like nature of trained immunity.

Collectively, in order to bridge innate and adaptive immunity and affect health and disease, other cell‐specific adaptations rooted in reversible epigenetic inheritance and metabolic reprogramming enable innate cells to remember prior stimuli.

## 3 Molecular Mechanisms of Trained Immunity

Trained immunity confers enhanced responsiveness to subsequent challenges so as to bridge innate and adaptive immunity [49]. At its core, epigenetic inheritance establishes heritable chromatin modifications to prime proinflammatory and antimicrobial gene expression [50]. Metabolic reprogramming as a shift from oxidative phosphorylation to aerobic glycolysis provides bioenergetic and biosynthetic substrates for immune cell activation [51]. Moreover, transcriptional regulation integrates epigenetic and metabolic signals via transcription factors to orchestrate cytokine secretion and PRR upregulation [52]. The mechanisms synergistically mediate innate immune plasticity, whose dissection not only advances understanding of immune memory but also exploits translational potential for developing novel immunotherapies for infection, inflammation, and cancer.

### 3.1 Epigenetic Inheritance

Epigenetic inheritance refers to the production of heritable and reversible phenotypic changes without altering the DNA sequence, mainly including DNA methylation and histone modification during trained immunity [53]. Unlike innate immunity, a specific feature of trained immunity is that the body will produce a stronger immune response after receiving a second stimulus. Epigenetic inheritance as the core mechanism of trained immunity enables immune cells to acquire memory through histone modification and other ways [54]. Moreover, because epigenetic inheritance is reversible, it may be possible to avoid the negative effects of immune memory.

#### 3.1.1 DNA Methylation

DNA methylation plays an important role in trained immunity [55]. The classical DNA methyltransferases (DNMTs) such as DNMT1, DNMT3A, and DNMT3B mediate DNA methylation modification [56]. The ten‐eleven translocation proteins function as erasers of DNA methylation mediate demethylation by oxidizing 5‐methylcytosine to 5‐hydroxymethylcytosine, 5‐formylcytosine, and 5‐carboxylcytosine [57]. Enhanced replication containment is observed in monocyte‐derived macrophages of individual subgroups inoculated with BCG [58]. Differential DNA methylation patterns stable for BCG can be observed in PBMCs isolated from inoculators but not in control group [59]. Gene ontology analysis that promoters with altered DNA methylation patterns were strongly enriched in genes belonging to the immune pathway in responders but not in control group. Bioinformatics analysis that differentially methylated genes were enriched in actin regulatory pathways in BCG inoculators, indicating differences in phagocytosis and suggesting that the phagocytosis function of immune cells is more effectively enhanced to kill pathogens [60].

#### 3.1.2 Histone Modification

Histone modifications, particularly decreased H3K27 acetylation, drive epigenetic inheritance predominantly during monocyte‐to‐macrophage differentiation, while dynamic changes are observed at both promoters and distal regulatory elements [61, 62]. Furthermore, the recent studies showed more intuitively the role of epigenetics in trained immunity. For instance, the inhibition of histone methyltransferases with 5′‐deoxy‐5′‐methylthioadenosine or inhibition of histone acetyltransferases with epigallocatechin‐3‐gallate drastically inhibited the training of monocytes [63, 64].

As monocytes develop into macrophages, distal regulatory elements acquire H3K27ac, which typically acquires H3K4me1 [65]. The process that monocytes develop into macrophages is usually accompanied by the appearance of H3K4me1, and the distal regulatory elements acquire H3K27ac. Poststimulus removal, H3K27ac decays while H3K4me3 stably accumulates on chromatin, sustaining immune gene activation and epigenetic memory for trained immunity [66]. In the initial phase of LPS stimulation‐induced trained immunity, the drastic changes of long‐term HSCs with a rapid increase in myeloid differentiation in the short term and a return to normal levels are caused by a single LPS exposure. However, it does not mean that immune memory is not produced. In fact, LPS training appears to epigenetically inscribe a long‐term cryptic memory of lineage bias with transcriptional silence. LPS‐exposed HSCs maintained access to many genes through C/EBPα to activate myeloid lineage differentiation more quickly upon secondary stimulation.

Similarly, after BCG vaccination, whole‐transcriptome analysis of purified HSPCs showed the upregulated expression of genes associated with bone marrow and granulocyte lineage initiation, indicating that healthy vaccinated individuals after vaccination also have biased myeloid development. Ostini et al. [67] describes this phenomenon in terms of potential enhancers. As for initial stimulation and signaling network activation, inactive and unlabeled enhancers are modified with transcription factors, and transcription factors acutely acquire typical histone modifications associated with the active enhancer region. However, especially as the H3K4me1 marker persists, many of the enhancers do not return to latent state after the stimulus is removed. With immune cells restimulated, a faster and more powerful enhancer‐mediated inflammatory response can be achieved. It may be one of the mechanisms by which trained immunity acquires memory.

### 3.2 Metabolic Reprogramming

Metabolic reprogramming is an integral part of trained immunity. Metabolic reprogramming endows immune cells with new functions via changing the normal physiological activities. Energy supply is indispensable to the activities of immune cells [68]. The tricarboxylic acid (TCA) cycle also undergoes modifications during trained immunity. Some TCA cycle intermediates (such as citrate and succinate) with pleiotropic effects accumulate in trained immune cells to act as signaling molecules and modulate epigenetic inheritance. When stimulated by different environments, immune cells change metabolism to meet energy needs. Therefore, metabolic reprogramming associated with trained immunity need to be discussed.

#### 3.2.1 Regulation of Glycolysis

The regulation of glycolysis associated with the activation of the mammalian target plays a central role in coordinating cell growth, metabolism, and immune function. The Akt/mTOR/HIF‐1α pathway has been identified as a central regulator of glucose metabolism for trained immunity [69]. Under the action of BCG and β‐glucan, AKT phosphorylation, mTOR, and HIF1α is further activated to induce increased glycolysis. In order to promote glycolysis, Akt/mTOR‐induced miR‐9‐5p targeting IDH3α reduces α‐ketoglutaric acid levels to stabilize HIF‐1α [70]. For instance, monocytes that have undergone β‐glucan training exhibit a series of changes, such as a decrease in basal oxygen consumption, an increase in glucose consumption, a decline in mitochondrial electron transport chain capacity, and a significant Warburg effect. Compared with oxidative phosphorylation, aerobic glycolysis produces less energy, but it generates lactic acid to synthesize amino acids, nucleotides, and other components necessary to meet the needs of active and well‐trained immune cells. Monocytes trained by BCG or oxidized low‐density lipoprotein (Ox‐LDL) showed increased glycolysis with an accompanying increase in oxygen consumption. Hence, regulation of glycolysis in metabolic reprogramming associated with trained immunity is worthy of attention.

#### 3.2.2 Altered Lipid Metabolism

Studies have shown that lipid metabolism is an important part of trained immunity and is involved in inflammation induced by immune training [71]. The cholesterol biosynthesis pathway, especially mevalonate synthesis, is one of the most upregulated pathways in bone marrow cell progenitors from β‐glucan‐trained mice. The methanoic acid as a metabolite mediates the activation of inflammatory pathways in trained immunity. In patients with high immunoglobulin D syndrome, monocytes accumulate mevalonate and develop a trained immunophenotype [72]. The mevalonate pathway responsible for cholesterol synthesis is upregulated in trained immune cells. With some studies suggesting a switch from long‐chain fatty acid oxidation to short‐chain fatty acid oxidation in certain conditions, fatty acid oxidation may also be altered [70]. The reprogramming in lipid metabolism contribute to the enhanced function and survival of trained immune cells.

#### 3.2.3 Amino Acid Metabolism

The reprogramming in amino acid metabolism has been shown to regulate function of immune cells [73]. Using classical biochemistry techniques, an increase in aspartate and glutamate metabolism in trained immune cells was observed, which provide a substrate for the TCA cycle. Fumarate is an intermediate metabolite of the TCA cycle, and a competitive inhibitor of several α‐ketoglutarate‐dependent dioxygenases, including histone demethylase. Fumarate as a critical role in DNA repair increases the level of dimethylation of histone H3 by inhibiting lysine demethylase 2B (KDM2B) activity, which is key to epigenetic trained immunity [74]. In addition to fumarate, multiple amino acid metabolic products are involved in the regulation of trained immunity. Therein, itaconate as metabolic product of the TCA cycle has been shown to have multiple anti‐inflammatory effects in macrophages via inducing immune tolerance. In order to promote succinate dehydrogenase (SDH) accumulation and contribute to the production of trained immunity, β‐glucan‐induced trained immunity reduces itaconate production by inhibiting immune‐responsive gene 1 [68]. Investigating amino acid metabolism in trained immunity provides novel insights into the molecular basis of innate immune plasticity and offers potential targets for therapeutic modulation of immune responses in health and disease.

### 3.3 Transcriptional Regulation

Transcriptional regulation as the molecular backbone of trained immunity integrates initial stimuli with long‐term epigenetic memory to shape innate immune responses [75]. By deciphering the crosstalk between histones and DNA methylation, the protective effects of trained immunity are harnessed while its pathological consequences are mitigated. The knowledge not only advances understanding of innate immune biology but also paves the way for innovative therapies targeting infectious diseases, chronic inflammation, and cancer.

#### 3.3.1 Activation of Transcription Factor

The activation of multiple transcription factors in innate immune cells are triggered training stimuli [76]. Therein, nuclear factor‐κB (NF‐κB) is rapidly activated in response to various immune stimuli [77]. In the context of trained immunity, NF‐κB activation leads to the transcription of genes encoding proinflammatory cytokines, chemokines, and antimicrobial peptides. Other transcription factors, such as IFN regulatory factors and activator protein 1, are also activated to contribute to the transcriptional reprogramming of innate immune cells [78]. These transcription factors often work in concert to regulate own expression via binding to specific promoter and enhancer regions of target genes.

#### 3.3.2 Long Noncoding RNA

Long noncoding RNA (lncRNA) was previously considered a nonsense product of transcription but was recently found to play an important role in acting as a transcriptional regulator and regulating epigenetic genes [79]. It can regulate gene transcription by regulating the three‐dimensional structure of chromatin. Fanucchi et al. [80] found that three‐dimensional chromatin topology allowed immune genes to make chromosomal contact with lncRNA and defined them as immune gene‐primed lncRNA. LncRNA transcribed within these structures direct the WDR5/MLL1 complex to target genes to initiate epigenetic modifications. Typical Immune gene‐initiated lncRNA, such as upstream master lncRNA of the inflammatory chemokine locus (UMLILO), promotes the epigenetic initiation of H3K4me3 through a chemokine promoter [81]. The mechanism among several trained immune genes, such as *IL6* and *CSF1*, is shared.

Posttranscriptional modification is a series of processes that modify RNA molecules after gene transcription. Posttranscriptional modifications have been found in all RNAs, including transfer RNA, ribosomes RNA, and messengers RNA (mRNA), as well as various short and lncRNAs [82]. According to the MODOMICS database, more than a hundred RNA modifications have been identified, including 5‐methylcytosine, N6, 2′‐O‐dimethyladenosine, and N4‐acetylcytidine [83]. Based on the improvement of sequencing technology, the understanding of RNA modification detection has been deepened. M6a as one of the most widely used RNA modification methods has been found to play an important role in tumor immunity. N6‐Methyladenosine (m6a), a double‐edged sword in trained immunity, can promote and alleviate tumor progression. METTL3 promotes tumor proliferation in bladder cancer via m6a [84]. N6‐methyladenosine (m6A) methylation and iron death contribute to the immune escape of lncRNA in hepatocellular carcinoma [85]. In addition, m6a associated with the prognosis of various tumors promotes the killing of tumor cells by regulating immune cells. The trained immunity acquires memory through epigenetic inheritance, such as histone modifications. Hence, lncRNA sustains proinflammatory or antimicrobial states via modulating epigenetic inheritance, rewiring metabolic reprogramming, and fine‐tune signaling cascades.

**FIGURE 2:**
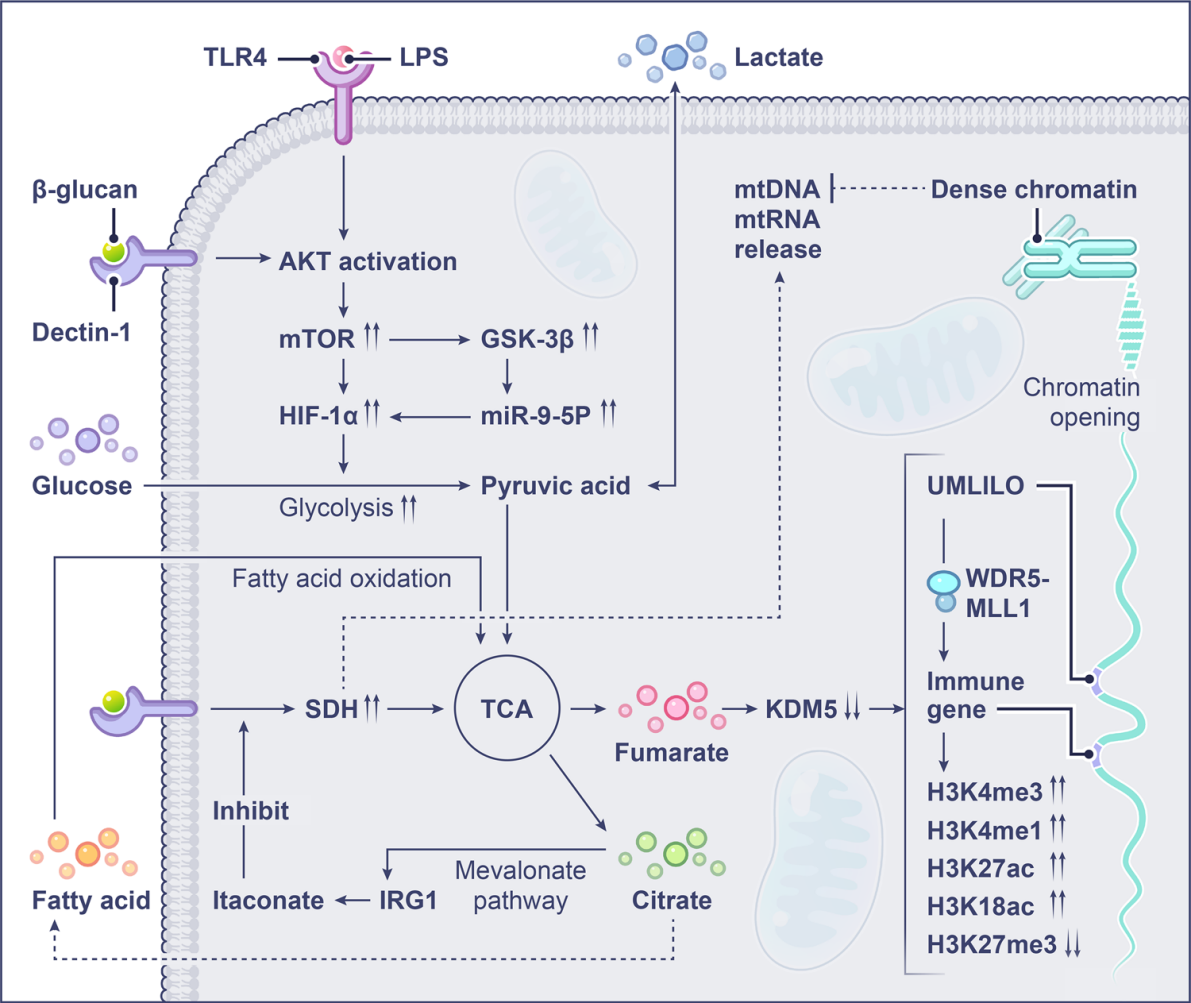
Trained immunity and its regulatory mechanisms. Trained immunity enables memory formation in innate immune cells through metabolic reprogramming and epigenetic inheritance. Dectin‐1, upon recognizing and binding to β‐glucan, activates the AKT–mTOR–HIF‐1α pathway, leading to an increase in glycolysis. Glucose is metabolized to pyruvic acid, and simultaneously, fatty acid oxidation is inhibited by SDH. The TCA cycle is involved, with its metabolites such as fumarate and citrate playing important roles. Fumarate inhibits KDM5 enzymes like H3K4, increasing chromatin methylation. Citrate participates in the mevalonate pathway and also influences epigenetic regulation. Lactate induces mtDNA release and chromatin opening. UMLILO regulates epigenetics by increasing H3K4 methylation in the WDR5–MLL1 complex in chromatin, along with other histone modifications like H3K4me1, H3K27ac, H3K18ac, and H3K27me3, which are all involved in the regulation of immune genes and the overall process of trained immunity.

Above all, a complex cellular signaling pathway involves in the trained immunity. The pathway is initiated by the interaction of TLR4 with LPS and lactate. β‐Glucan activates Dectin‐1, leading to AKT activation and subsequent effects on mTOR and GSK‐3β. Glucose metabolism is affected by terms in the altered glycolysis and pyruvic acid levels. Fatty acid oxidation is inhibited to a decrease in SDH and an increase in fumarate. KDM5 and WRD5 proteins affected by fumarate lead to chromatin modifications, such as H3K4me3, H3K4me1, H3K27ac, H3K18ac, and H3K27me3. These modifications regulate immune gene expression. Additionally, the pathway involving the release of mtDNA and dense chromatin mRNA leads to chromatin opening and the regulation of UMLILO and WD‐MLL1 (Figure 2). The overall pathway highlights the intricate interplay between various cellular components and metabolic processes in modulating immune gene expression.

## 4 Pros and Cons of Trained Immunity

Trained immunity refers to the long‐term functional remodeling of innate immune cells, such as monocytes, macrophages, and NK cells, through mechanisms like epigenetic inheritance and metabolic reprogramming in response to pathogens, vaccines, or environmental stimuli. The remodeling enables these immune cells to react more rapidly and robustly upon subsequent encounters with the same stimuli. Its characteristic play dual roles in health and disease.

### 4.1 The Dual Roles in Infection

In infection‐related diseases, trained immunity operates as a critical early defense against pathogens but can also drive tissue damage, chronic inflammation, and autoimmune complications. In order to highlight future directions for harnessing benefits and mitigating harms, the dual roles were summarized from protective mechanisms, pathogenic risks, and clinical implications.

#### 4.1.1 Protective Roles

Trained immunity emerges as a cornerstone of innate immune defense, particularly in the early stages of infection or in populations with impaired adaptive immunity. Trained innate cells with heightened effectors enables pathogens eliminated faster and more efficient. After trained by mycobacterium tuberculosis or LPS, immune cells display increased phagocytic capacity, ROS production, and secretion of proinflammatory cytokines and antimicrobial peptides. For example, trained macrophage from BCG‐vaccinated individuals shows enhanced killing of *Mycobacterium tuberculosis* via upregulation of the NLRP3 inflammasome [86]. Additionally, trained immunity‐induced epigenetic upregulation of cytotoxic molecules and cytokine receptors strengthen the ability to lyse virus‐infected cells and secrete IFN‐γ to inhibit viral replication. A phenomenon that trained immunity confers heterologous protection against unrelated pathogens is exploited by live attenuated vaccines to broaden immune coverage. Taking BCG vaccination as the most well‐characterized example, trained immunity in ILCs and macrophage via TLR2/4 signaling was induced to reduce mortality from pathogens and improve outcomes in viral infections by enhancing IFN‐γ production [87, 88]. Influenza vaccine‐induced trained immunity (VITI) in DCs amplifies cross‐reactive T cell responses to heterologous influenza strains, reducing disease severity [89]. Hence, its protective effects are mediated by the above key mechanisms.

#### 4.1.2 Pathogenic Risks

While trained immunity is protective, its persistent proinflammatory state can lead to unintended harm, including tissue damage, chronic inflammation, and autoimmune disease. Trained immunity‐mediated hyperactivation of innate cells drives uncontrolled cytokine secretion, exacerbating organ injury. In sepsis, trained monocytes with overexpressing TLR4 leads to increased mortality via secreting massive IL‐6 and TNF‐α [90]. In severe COVID‐19, trained immunity‐induced epigenetic reprogramming of alveolar macrophages promotes sustained IL‐1β secretion to contribute to pulmonary edema and acute respiratory distress syndrome [91]. Trained immunity can disrupt immune tolerance by promoting self‐reactive innate responses. trained immunity in monocytes induced by chronic exposure to porphyromonas gingivalis leads to persistent production of anticitrullinated protein antibodies and synovial inflammation via epigenetic upregulation of cytokine genes. Trained immunity in DCs is triggered by viral infections to enhance type I IFN production and drive autoantibody formation against nuclear antigens. In some chronic infections, trained immunity paradoxically impairs pathogen clearance by inducing innate cell exhaustion or metabolic dysfunction [92]. Persistent *Mycobacterium tuberculosis* infection trains macrophages to adopt a hypofunctional state. It is characterized by reduced ROS production and increased lipid droplet accumulation, allowing bacteria to evade clearance and establish latency [93]. Moreover, trained immunity in NK cells from HIV‐infected individuals contributes to viral persistence via reducing cytotoxicity and IFN‐γ secretion due to chronic viral antigen exposure [94].

#### 4.1.3 Clinical Implications and Future Directions

The bifunctional property of trained immunity in infection underscores its potential as a therapeutic target. Harnessing protective and mitigating pathogenic trained immunity emerge as two key strategies. Development of trained immunity‐modulating vaccines to enhance innate defense against emerging pathogens or in immunocompromised populations [95]. Additionally, use of epigenetic modifiers or metabolic regulators to suppress excessive inflammation in cytokine storm syndromes or autoimmune diseases [96].

### 4.2 The Dual Roles in Inflammation

Inflammation is a central pathological driver of cardiovascular diseases, mediating its initiation, progression, and acute complications through persistent immune cell infiltration and proinflammatory signaling [97]. Cardiovascular diseases are currently a major global health burden [98]. With the ageing of society and the increase in risk factors, the incidence of cardiovascular diseases has been high worldwide [99]. Trained immunity, a cornerstone of innate immunology, describes the long‐term functional reprogramming of innate immune cells induced by stimuli such as microbial components, metabolic byproducts, or environmental factors. Unlike adaptive immunity, which relies on antigen specificity, trained immunity is driven by epigenetic and metabolic reprogramming, enabling innate immune cells to mount enhanced responses to subsequent challenges. A defining feature of the process is its dichotomous role in inflammation. While it protects against infections and malignancies by priming rapid, effective immune responses, it can drive chronic inflammation and autoimmune diseases when dysregulated. Based on the dual characteristics of inflammation‐induced training immunity, protective roles, pathogenic risks, and clinical application potential are elaborated.

#### 4.2.1 Protective Roles

Trained immunity refers to a long‐term functional memory exhibited by innate immune cells via epigenetic reprogramming and metabolic remodeling, enabling enhanced responsiveness to subsequent stimuli [100]. Trained immunity plays a pivotal protective role by multidimensionally regulating innate/adaptive immunity, lipid metabolism, and endothelial function. For example, β‐glucan or BCG‐induced trained immunity promotes the expression of anti‐inflammatory genes, and suppresses hyperactivation of proinflammatory genes via modifications of activating histone marks and enhancer marks. The epigenetic changes enable long‐term functional regulation with persistence for weeks to months. In order to inhibit excessive activation of the NF‐κB pathway and reduce proinflammatory cytokine secretion, trained innate cells enhance ATP generation through aerobic glycolysis and produce metabolic byproducts. For instance, the glycolytic intermediate phosphoenolpyruvate inhibits the AKT pathway, diminishing macrophage uptake of Ox‐LDL and limiting foam cell formation [101]. Additionally, monocytes and macrophages are the predominant inflammatory cells in atherosclerotic plaques. Trained immunity inhibits plaque progression via reprogramming these cells from a proinflammatory (M1) to an anti‐inflammatory (M2) phenotype. Trained circulating monocytes, particularly the CD14⁺CD16⁺ subset, exhibit a greater propensity to differentiate into M2 macrophages. The proportion of CD14⁺CD16⁺ on monocytes' surface and the modification level of H3K4me3 can be used as predictive indicators for the risk of AS. Through phagocytosis of Ox‐LDL, IL‐10 secretion, and transforming growth factor‐β (TGF‐β), the monocytes reduce the risk of plaque rupture via facilitating intraplaque lipid clearance and enhance fibrous cap stability. The phagocytic capacity of trained macrophages is augmented via upregulation of scavenger receptors, while reverse cholesterol transport activity is enhanced through cholesterol efflux to high‐density lipoprotein [102]. Collectively, the changes reduce foam cell accumulation.

Trained immunity regulates the balance of adaptive immunity via functional remodeling of DCs, thereby suppressing atherosclerosis (AS)‐associated autoimmune responses. DCs exhibit improved capacity to present self‐antigens via upregulation of MHC‐II molecules and costimulatory molecules, promoting the activation of regulatory T (Treg) cells. Treg cells suppress the proinflammatory activity of Th1/Th17 cells via secretion of IL‐10 and TGF‐β. Treg cells activation reduces endothelial dysfunction via inhibiting cytotoxic T cell (CTL) attack on endothelial cells. Additionally, B lymphocytes production of anti‐Ox‐LDL antibodies is suppressed to attenuate inflammation induced by immune complex deposition in plaques.

AS is a chronic inflammatory lipid metabolic disease, while endothelial injury is the initiation of AS. Trained immunity protects endothelial cells via circulating factors or exosomes, reducing monocyte adhesion and infiltration. Trained monocytes secrete granulocyte–macrophage colony‐stimulating factor and vascular endothelial growth factor (VEGF), which promote endothelial cell proliferation and repair. Additionally, in order to improve endothelial dilatory function and reduce Ox‐LDL uptake, NO production is enhanced via activation of endothelial NO synthase [103]. To reduce monocyte adhesion, a critical step in plaque formation that trained immunity reduces the expression of intercellular adhesion molecule‐1 and vascular cell adhesion molecule‐1 on endothelial cell surfaces [104]. Trained immunity regulates lipid metabolism pathways to reduce intraplaque cholesterol deposition and promote cholesterol excretion. Additionally, lipoprotein lipase activity is enhanced to promote triglyceride hydrolysis and reducing very low‐density lipoprotein production. With Ox‐LDL being the key antigen driving plaque formation, trained immunity reduces NADPH oxidase activity, diminishing ROS production and thereby inhibiting LDL oxidation [105].

BCG or β‐glucan and other training immune inducers can activate the innate immune system and reduce plaque inflammation. Drugs targeting glycolysis or epigenetic enzymes can optimize the anti‐inflammatory function of the training immune system. Hence, the protective effect of trained immunity provides a new target for the treatment of AS.

#### 4.2.2 Pathogenic Risks

AS is one of the main causes of cardiovascular diseases [106, 107]. A sufficient evidence that AS is a chronic inflammatory disease of blood vessel walls associated with LDL accumulation and immune cells such as T cells, B cells, and macrophages [108]. Modified lipoproteins, especially Ox‐LDL, can act as training stimuli for innate immune cells, particularly monocytes and macrophages in the arterial wall. Trained macrophages in the atherosclerotic plaque show enhanced production of proinflammatory cytokines, such as IL‐6 and TNF‐α, and increased lipid accumulation. The chronic inflammation can lead to the recruitment of more immune cells, the formation of foam cells, and the progression of atherosclerotic lesions. Additionally, lifestyles, such as a high‐fat diet, smoking, and lack of exercise, can also contribute to the activation of trained immunity in the context of AS.

Recently, studies have found that a variety of bacterial and viral infections increase the risk of AS [109, 110]. Invasion of pathogens triggers trained immunity, providing strong protection against reinfection, but trained macrophages show atherogenic phenotypes in cytokine production and foam cell formation [111]. Trained immune cells induce a long‐term proinflammatory phenotype through metabolism and epigenetics, thereby inducing a long‐term inflammatory response in the blood vessel wall [112]. Monocyte‐differentiated macrophages play a key role in the process [113]. Ox‐LDL is an endogenous atherogenic irritant. In research in vitro, monocytes increased mRNA expression and protein formation by exposing isolated human monocytes to Ox‐LDL. The process is associated with increased trimethylation of lysine 4 at histone 3 in the promoter region [114]. Meanwhile, immune‐trained monocytes promote foam cell formation. These results suggest that Ox‐LDL can induce a persistent proatherosclerotic macrophage phenotype by epigenetic histone modification of monocytes. Changes in energy metabolism are a common method of trained immunity. With the expression of genes related to mitochondrial metabolism upregulated, the mitochondrial TCA cycle in mononuclear macrophages induced by Ox‐LDL is the most upregulated pathway. Monocytes from patients with symptomatic AS showed increased relative mRNA expression of the glycolytic rate‐limiting enzyme hexokinase‐2 and 6‐phosphofructose‐2‐kinase/fructose‐2, 6‐bisphosphatase 3, which significantly increased glycolytic flux [115]. The feature is similar to other methods of inducing trained immunity, such as BCG‐induced trained immunity.

AS also leads to the trained immunity of nonimmune cells such as endothelial cells and smooth muscle cells. Sohrabi et al. [116] induced human aortic endothelial cells with Ox‐LDL, and its proinflammatory memory was restimulated with a TLR2 agonist. In addition to the production of inflammatory cytokines, in order to increase glucose consumption, lactate production, and epigenetic modification of inflammatory gene promoters, characteristic epigenetic and metabolic reprogramming of active human aortic endothelial cells are caused by Ox‐LDL via activation of mTOR–HIF1α signaling [117]. Epigenetic and metabolic mechanisms of innate immunity trained in monocytes can be duplicated in smooth muscle cells [118]. The changes in mononuclear macrophages induced by Ox‐LDL suggest that AS leads to trained immunity, which may be one of the reasons for the long‐term chronic inflammation in AS patients [119].

#### 4.2.3 Potential for Clinical Application

Inflammation‐induced trained immunity, referring to the long‐term epigenetic reprogramming of innate immune cells after exposure to inflammatory stimuli, has shown great potential for clinical application via endowing cells with enhanced responsiveness to subsequent stimuli [100]. In infectious diseases, trained immunity can be harnessed to enhance the body's ability to fight off pathogens. By priming innate immune cells through specific inflammatory signals, the immune response against various infections is boosted to reduce the severity and duration of the disease. In vaccination, the concept of trained immunity provides a new perspective. It may allow for the development of vaccines that not only stimulate adaptive immunity but also activate trained immunity, leading to more comprehensive and long‐lasting protection. In addition, in some noninfectious inflammatory diseases, understanding and modulating trained immunity could offer novel therapeutic strategies. For example, in autoimmune diseases, proper regulation of trained immunity may help to rebalance the immune system and alleviate the symptoms. Overall, the potential for clinical application of inflammation‐induced trained immunity is vast, and further research in the area holds promise for health.

**FIGURE 3:**
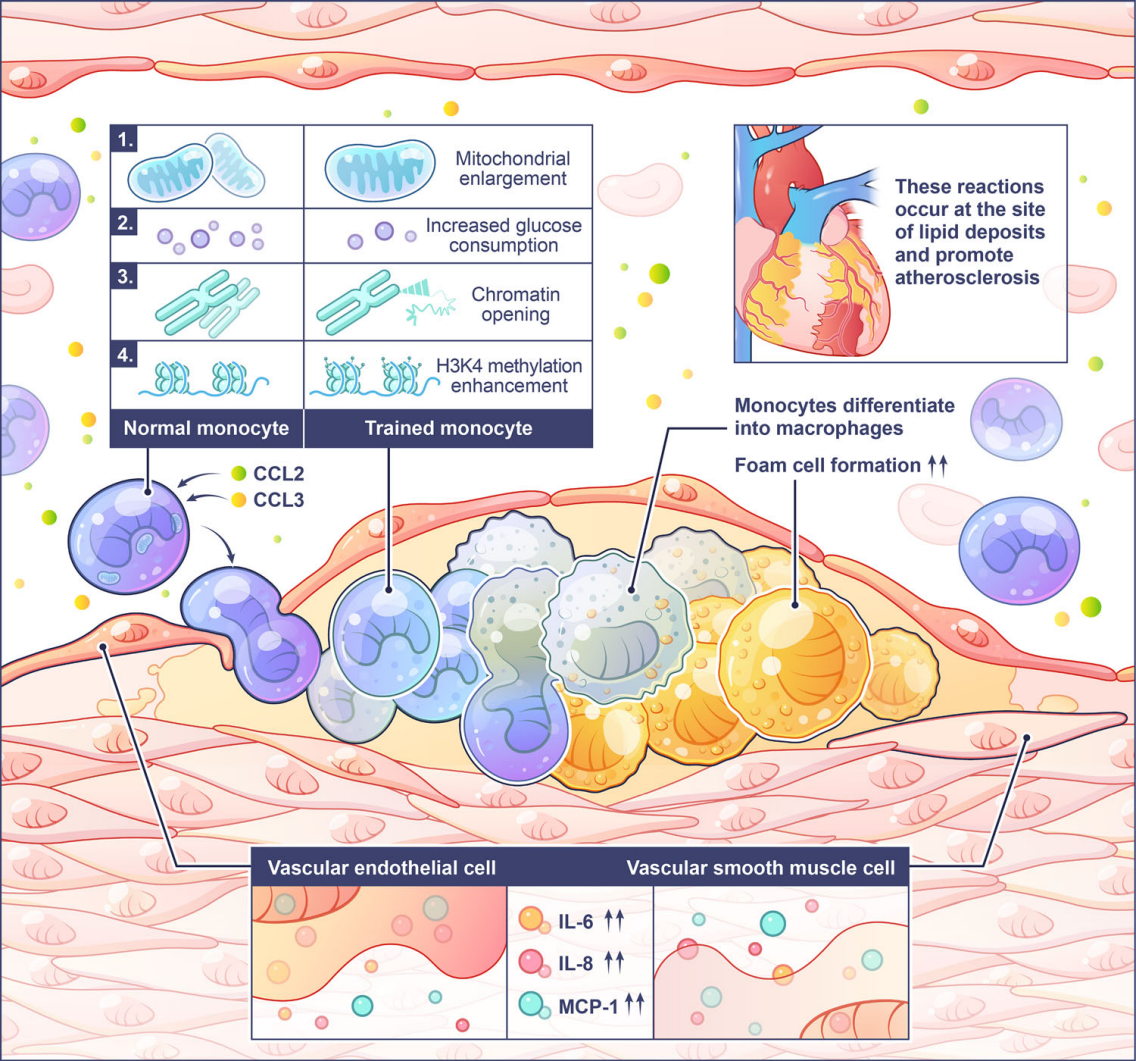
Trained immunity promotes atherosclerosis. In the early stage of AS, vascular endothelial permeability increases at the injured site, and Ox‐LDL is deposited subcutaneously in the blood vessels. Monocytes accumulate at the Ox‐LDL deposition site under the action of chemokines such as CCL2 and CCL3. Subsequently, monocytes are trained under the action of Ox‐LDL, with enlarged mitochondria, increased glucose consumption, enhanced glycolysis, and epigenetic inheritance such as methylation. After training, monocytes differentiate into macrophages under the endoderm. Their ability to phagocytose lipids is enhanced, resulting in an increase in foam cells. Endothelial cells and vascular smooth muscle cells can also be trained under the action of Ox‐LDL, and the secretion of IL‐6, IL‐8, MCP‐1, and other cytokines increases, further promoting vascular inflammation, resulting in an increase in foam cells.

To sum up, the core pathology of AS, accompanied by monocyte recruitment, vascular endothelial damage, and plaque formation, involves macrophages taking up Ox‐LDL to form foam cells (Figure 3). Trained immunity modulates the functional plasticity to exert a bifunctional role in AS. It suppresses foam cell formation and retards plaque progression by enhancing anti‐inflammatory metabolic reprogramming and epigenetic regulation in macrophages. And it promotes monocyte recruitment to the vascular wall and exacerbates plaque instability via sustained proinflammatory signaling and matrix degradation.

### 4.3 The Dual Roles in Tumors

It is widely accepted that tumors are related to the immune response. Inflammation, one of the hallmarks of tumors, can affect the occurrence and development of tumors by influencing the fate of various cells and reshaping the ecological niche of the tumor microenvironment (TME) [120]. Tumor‐associated macrophages (TAMs) can be trained by tumor‐derived factors, such as cytokines, chemokines, and exosomes. Trained TAMs associated with immunosuppression, angiogenesis, and tumor progression often acquire an M2‐like phenotype. Cytokines produced by it can inhibit the function of CTLs and NK cells, allowing the tumor cells to evade the immune system. Additionally, trained TAMs can promote angiogenesis by producing VEGF, which is essential for tumor growth and metastasis [121]. Hence, the factors secreted by trained tumor‐related immune cells without antitumor ability accelerate tumor metastasis via promoting tumor angiogenesis and immune suppression.

#### 4.3.1 Protective Roles

Trained immunity also inhibits tumorigenesis. Due to the rich heterogeneity of macrophages, macrophages are not always tumor‐promoting. Macrophages trained with whole β‐glucan granules showed increased reactivity not only to LPS but also to TNF‐α tumor‐derived factors [122]. Enhanced sphingolipid synthesis was followed by S1P accumulation in macrophages [123]. In addition to the increased Drp‐1 activation and mitochondrial fission, S1P resulted in increased mtROS production and cytotoxicity to tumor cells through inhibition of tumor metastasis and prolonged survival of mice in multiple metastatic models [124]. β‐glucan stimulates trained bone marrow cells to flow into the pancreas via CCR2‐dependent flow without damaging normal pancreatic tissue [125]. The cultivated bone marrow‐derived cells will differentiate into specific proinflammatory monocytes. These cells are capable of effectively killing tumor cells due to enhanced phagocytic capabilities and cytotoxic effects mediated by ROS. After recovery from influenza A virus (IAV) infection, the lungs develop long‐lasting antitumor immunity [25]. Resident AM in tissues exposed to IAV has enhanced phagocytotoxic and tumor cytotoxic functions due to the basic characteristics of trained immunity. Trained but not naive AMs are resistant to the tumor‐associated immunosuppressive microenvironment at the transcriptional and epigenetic levels. β‐Glucan induces the training of granulocyte production in BM, while type I IFN signaling is involved in trained granulocyte production and mediates the rewiring of neutrophils to antitumor phenotypes [126]. ScATAC‐seq analysis also confirmed that epigenetic changes were characteristic of trained granulocytes. Based on continuous in‐depth research on tumor immunity, related immunotherapies have been constantly introduced. The BCG vaccine has been widely used to treat cancer due to its safety and its role in inducing trained immunity. Intravesical BCG infusion has been used as an adjunctive therapy for nonmuscular invasive bladder cancer, and it can effectively reduce tumor recurrence and progression [127]. In addition, trained immunity is also part of the treatment. Enhancing trained immunity can reduce the occurrence of tumors by various means, such as irreversible electroporation which enhanced β‐glucan‐induced training of innate immunity for pancreatic ductal adenocarcinoma, and nanobiologic therapy that inhibited tumor growth and enhanced checkpoint inhibition to control tumor progression [10].

#### 4.3.2 Pathogenic Risks

To fully elucidate the role of trained immunity in promoting tumorigenesis, the origins, phenotypic reprogramming, functional regulation, and clinical implications of TAMs are expanded [128]. Below is a structured amplification of the original content, integrating mechanistic details, epigenetic mechanisms, and translational relevance. Trained immunity drives tumor progression by reprogramming TAMs [129]. TAMs originate primarily from circulating Ly6C^+^ monocytes, which are recruited to the TME via the CCL2–CCR2 axis [130]. Tumor cells and stromal fibroblasts secrete protein called CCL2, which binds to CCR2 on monocytes, directing their transendothelial migration into the hypoxic, acidic TME [131]. Once infiltrated, monocytes undergo profound phenotypic and functional changes mediated by trained immunity, transforming into protumorigenic TAMs that sustain tumor growth, angiogenesis, and immune evasion.

A key discovery in trained immunity‐mediated tumorigenesis is the differentiation of TAMs into a Bcl6^+^ CD11b^+^ F4/80^+^ Ly6C^−^ phenotype, which underpins their long‐term protumor activity [132]. With epigenetic and metabolic reprogramming as core mechanisms, the process is regulated by a TLR4/mTOR/AKT–Bcl6 signaling axis. The TME is rich in endogenous TLR ligands, including HMGB1, heat shock proteins, and oxidized lipids [133]. The ligands activate the NF‐κB/MAPK pathway and upregulate the transcription factor Bcl6 via binding to TLR4 on monocytes [134]. Bcl6, a master regulator of TAM polarization, suppresses proinflammatory genes and activates protumor genes, shifting monocytes toward an “M2‐like” protumor phenotype. Concurrent with TLR4 activation, the mTOR/AKT pathway is triggered by TME‐derived growth factors and nutrient stress [135]. With the pathway promoting glycolytic metabolic reprogramming, monocytes upregulate glucose transporter 1 and lactate dehydrogenase A, increasing lactate production to support rapid proliferation [136]. Increased H3K4me3 at the Bcl6 promoter enhances its transcription, while decreased DNA methylation at protumor gene promoters stabilizes their expression [137, 138]. Together, with these changes locking monocytes into a trained state, they retain memory of initial TLR4 stimulation and rapidly reactivate protumor responses upon re‐exposure to TME signals.

Bcl6^+^TAMs further differentiate into stem cell‐like memory macrophages (SMMs) via a Bcl6‐driven transcriptional program [139]. In order to allow rapid reactivation of protumorigenic cytokine secretion and metabolic activity upon re‐exposure to TME ligands, markers of stem cells and the Wnt/β‐catenin pathway are not only expressed to maintain proliferation and enable long‐term survival in the TME, but also retain epigenetic memory of TLR4 stimulation. The memory ensures stable protumor activity, even in the absence of continuous TLR4 stimulation.

Trained TAMs drive tumor progression [140]. In order to form leaky tumor blood vessels that supply nutrients and remove waste, SMMs secrete VEGF‐A and angiopoietin‐2 to promote endothelial cell proliferation and vascular sprouting [141]. They create paths for tumor cells to invade surrounding tissue via producing MMP2/9 to degrade the extracellular matrix. SMMs also secrete CXCL12 to attract tumor cells to lymphatic vessels, facilitating metastasis. SMMs release TGF‐β and IL‐10 to suppress CD8^+^ T cell and NK cell activity, while expressing PD‐L1 to inhibit T cell proliferation. Tregs cells are recruited to dampen antitumor immunity via CCL22 [142].

#### 4.3.3 Clinical Implications and Future Directions

**FIGURE 4:**
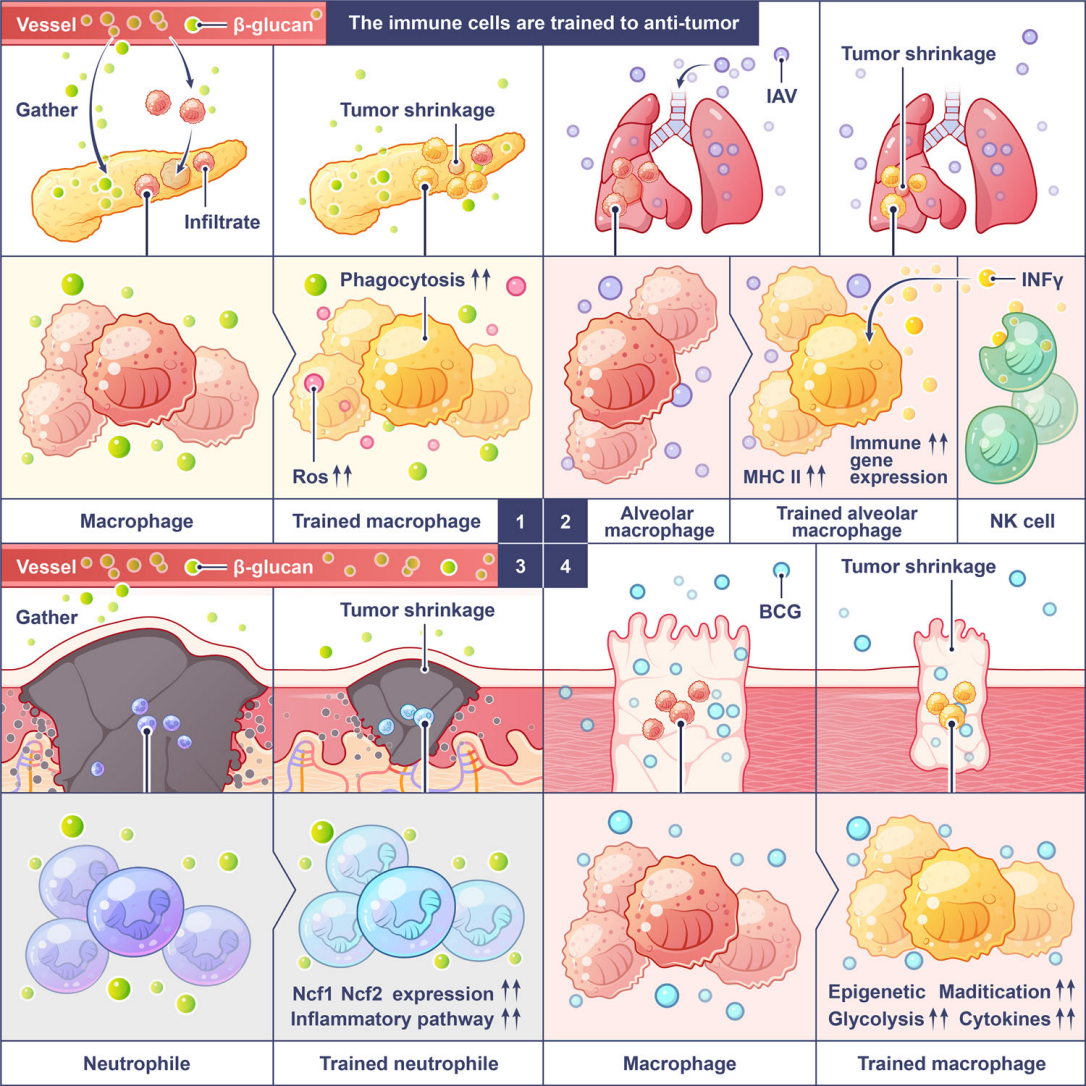
Enhanced trained immunity facilitates the eradication of tumors. Tumors are closely related to the immune system. Trained immunity is expected to be a new tumor therapeutic strategy based on increasing the tumor killing capacity of innate immune cells. (1) β‐Glucan accumulates in the pancreas, stimulating the infiltration of bone marrow immune cells and inducing trained immunity. These trained cells differentiate into specific proinflammatory macrophage/monocyte populations with enhanced phagocytosis and ROS‐mediated cytotoxicity to kill tumor cells effectively. (2) Influenza A virus (IAV) infection induces the trained immunity of AM cells in the lung, which requires the involvement of IFNγ and NK cells. Trained AM cells upregulate the expression of transcripts associated with antitumor function, including antigen processing and presentation, phagocytosis, and Toll‐like receptor signaling pathways, and enhance mitochondrial oxidation, leading to improved phagocytosis and tumor cytotoxicity. (3) β‐glucan induces trained immunity in neutrophils and granulocyte–monocyte progenitors (GMPs) in mouse bone marrow, and type I IFN signaling is involved in trained granulocyte production and the antitumor phenotype of neutrophils. After training, neutrophils exhibited antitumor activity in a ROS‐dependent manner by activating NCF1 and NCF2, genes related to ROS production. (4) BCG is injected into the bladder and absorbed by tumor cells to initiate an immune response. The increase in inflammatory factors released by the cells promotes the migration of monocytes to the bladder epithelium to become domesticated macrophages, thereby mediating the killing of tumor cells.

The role of trained TAMs in tumorigenesis has significant clinical relevance. The abundance of Bcl6^+^CD11b^+^F4/80^+^Ly6C^−^ SMMs in tumor tissues correlates with advanced tumor stage, increased metastasis, and poor overall survival in patients with colorectal cancer, breast cancer, and glioblastoma [139]. For example, a study from Henderson et al. [143] found that SMM density was an independent predictor. Targeting the TLR4/mTOR/AKT–Bcl6 axis is a promising strategy to reverse TAM polarization and inhibit tumor growth. By inhibiting monocyte activation, Bcl6 expression and glycolytic reprogramming, the formation of tumor‐promoting macrophages is blocked and their functions are reversed. Meanwhile, proinflammatory genes are activated to restore the antitumor activity of macrophages. The mechanism underly tumor regression reveals how β‐glucan and BCG initiate a multicellular coordinated antitumor cascade reaction through training immunity (Figure 4).

Trained immunity promotes tumorigenesis by inducing epigenetic, metabolic, and phenotypic reprogramming of TAMs, driving their differentiation into Bcl6^+^ SMMs with stem‐like properties and stable protumorigenic activity [144]. The mechanism highlights the critical role of innate immune memory in tumor progression and provides a framework for developing novel immunotherapies. Future research should focus on combining TAM‐targeted agents with checkpoint inhibitors or CAR‐T cells to enhance antitumor efficacy by reversing immune suppression in the TME [145]. As knowledge of trained immunity in tumor deepens, these strategies may transform the treatment of advanced tumors.

## 5 Trained Immunotherapy

The concept of trained immunotherapy represents a paradigm shift in oncology, harnessing the adaptive memory of immune cells to combat malignancies [146, 147]. Unlike conventional therapies, which indiscriminately target tissues, immunotherapies exploit the specificity of immune cells to recognize and eradicate pathogens [148]. Pioneering studies demonstrate that combining immune checkpoint inhibitors (ICIs) with chemotherapy enhances response rates and survival. Notably, prophylactic cancer vaccines have prevented several malignancies by protecting against cancer‐causing pathogens [149]. However, challenges persist, including cytokine release syndrome and TME suppression. Emerging strategies aim to refine immune cell persistence and safety through dose escalation or epigenetic inheritance. This evolving field promises not just incremental improvements but transformative cures, redefining the boundaries of precision medicine.

### 5.1 Trained Immunity‐based Therapy

Trained immunity‐based therapy (TIBT), a groundbreaking strategy leveraging innate immune memory, has emerged as a transformative alternative to traditional antigen‐specific vaccines, addressing unmet needs in health and disease [150]. Relevant trials highlight its versatility as a therapeutic target across infectious, inflammatory, and cancerous diseases (Table 2). While early results are encouraging, larger, longer‐term studies are needed to establish optimal dosing, patient selection, and safety profiles. The integration of trained immunity with existing therapies may revolutionize treatment paradigms by harnessing the untapped potential of innate immune system. Mechanically, by inducing epigenetic and metabolic reprogramming in innate immune cells via bioactive adjuvants, TIBT extends protective immunity beyond adaptive responses, offering broad‐spectrum and long‐lasting protection against diverse pathogens and malignancies.

**TABLE 2:** Registered clinical trials on trained immunity.

Types	Conditions	ClinicalTrials.gov ID	Phase	Intervention/treatment	References
Infection	BCG vaccination	NCT02175420	Completed, not applicable	Biological: BCG Biological: TFV	[151]
		NCT06628544	Early phase 1	Biological: BCG vaccination Drug: metformin is administered on the day 0 Other: placebo Drug: metformin is administered on the day 9 Drug: metformin is administered on the day 85	[152]
		NCT02259608	Completed, not applicable	Biological: BCG vaccine SSI	[153]
		NCT02114255	Completed, phase 2/3	Other: placebo Biological: BCG	[154, 155, 156, 157]
		NCT03296423	Completed, phase 4	Biological: vaccination Biological: placebo	[158]
		NCT04348370	Completed, phase 4	Biological: BCG vaccine Biological: placebo vaccine	[159, 160]
		NCT04417335	Unknown status, phase 4	Biological: BCG vaccine Biological: placebo vaccine	[155, 161]
		NCT04414267	Completed, phase 4	Biological: BCG vaccine Biological: placebo vaccine	[162]
		NCT04328441	Completed, phase 3	Drug: BCG vaccine Drug: placebo	[159, 163, 164]
		NCT05507671	Active, not recruiting, Phase 3	Biological: BCG (Bacillus Calmette–Guérin) vaccine Other: placebo	[165]
	COVID‐19	NCT04798677	Completed, not applicable	Dietary supplement: ABBC1 immunoessential Dietary supplement: placebo	N/A
		NCT04475081	Completed, phase 3	Biological: MMR vaccine	[166]
		NCT04646239	Completed	Diagnostic test: heterologous stimuli Diagnostic test: neutralization assay	[167, 168]
		NCT06341374	Recruiting	Biological: influvac tetra	[169]
		NCT05705180	Unknown status	Biological: recombinant adjuvanted zoster vaccine	[170, 171]
	HIV‐1‐infection	NCT04968717	Completed	Observation	[172]
		NCT06959563	Active, not recruiting, early phase 1	Biological: 9vHPV vaccine plus BCG vaccine mix for percutaneous use	N/A
	Depression	NCT03756246	Unknown status, phase 4	Biological: influenza vaccine	[173]
	Respiratory system abnormalities	NCT05802355	Completed, not applicable	Other: manual diaphragm release Other: control group	[174]
	BK polyomavirus	NCT06988072	Not yet recruiting	Other: blood sampling Other: blood sampling Other: blood sampling	[175]
	Influenza, Herpes Zoster	NCT05082688	Completed, phase 2	Biological: herpes zoster vaccination (Shingrix, GSK) Biological: influenza vaccine (Fluarix Tetra Northern Hemisphere 2021 or 2022, GSK) Biological: placebo	[155]
	Vaccine reaction	NCT06266754	Enrolling by invitation, phase 4	Biological: oral polio vaccine Other: placebo	[176]
	Acute respiratory infections (ARIs)	NCT06746259	Recruiting	Diagnostic test: rapid point of care test to detect host immune response in ARI	[177]
	Tropical infectious disease	NCT05722054	Unknown status	Other: diagnostic tests Other: equilibrate diet	[178]
Inflammation	Cardiovascular diseases	NCT05682456	Completed, not applicable	Dietary supplement: high‐fat shake Dietary supplement: reference shake	[179]
		NCT03172507	Completed	Observation	[180]
	Atherosclerosis	NCT03354156	Completed	Drug: statin treatment	[181, 182]
		NCT02817230	Completed	Drug: statin	
	Coronary artery disease	NCT05210725	Phase 4	Drug: rivaroxaban 2.5 mg oral table	[183]
		NCT06562309	Completed	Other: take feces and blood samples from patients	[184, 185, 186]
	Immune response	NCT05208060	Phase 1/2	Biological: MV130 Other: placebo	[187]
		NCT03240497	Completed, not applicable	Behavioral: cold exposure Behavioral: strength ventilation	[188, 189]
	Cholesterol variability	NCT05790499	Unknown status, not applicable	Drug: atorvastatin	[190]
	Systemic lupus erythematosus	NCT04570306	Completed	Drug: belimumab	[191]
	Fibromyalgia	NCT03441997	Unknown status, not applicable	Other: mind‐body exercise Other: light mobility exercises	[192]
	Gastrointestinal disease	NCT04498208	Completed, not applicable	Behavioral: physical prehabilitation Behavioral: stress reduction prehabilitation Behavioral: cognitive prehabilitation Behavioral: nutrition prehabilitation	[193]
	Chronic hepatitis b	NCT06047093	Recruiting, not applicable	Procedure: investigation of the intrahepatic compartment using the fine needle aspiration (FNA) technique Biological: creation of a serum biobank Other: FNA feasibility and acceptability questionnaire	[194]
	IgG4‐related disease	NCT06844864	Active, not recruiting	Observation	[195]
Cancer	Thyroid cancer, nonmedullary, colon carcinoma	NCT05280379	Active, not recruiting	Other: no intervention will take place	[51]
	Rectal cancer	NCT02403505	Active, not recruiting, phase 1	Biological: CEA protein antigen plus BCG vaccine mix for percutaneous use	N/A
	Cancer liver	NCT06786429	Not yet recruiting, phase 4	Biological: gardasil 9 vaccine Biological: hepatitis B virus vaccine (HBV) Behavioral: cognitive behavioral therapy	[196]

#### 5.1.1 Prevention of Infectious Diseases

The most compelling advantage of TIBT with a feature unattainable by conventional vaccines lies in its cross‐protective efficacy against unrelated pathogens. Preclinical studies have demonstrated that trained immunity‐based vaccines (TIBVs) can reduce mortality associated with influenza, bacterial sepsis, and fungal infections by priming innate cells to mount amplified cytokine responses and enhance phagocytosis upon restimulation. Clinical evidence, such as BCG's cross‐protective effects against nontuberculous infections, further underscores translational potential. However, in order to elucidate tissue‐specific memory mechanisms and validate long‐term safety profiles, challenges persist in optimizing adjuvant formulations.

Notably, BCG vaccine originally designed for tuberculosis has been shown to reduce childhood mortality from nontuberculous infections in clinical trials via training macrophages to mount faster, more robust cytokine responses. Novel TIBT formulations, such as β‐glucan‐adjuvanted influenza vaccines, have demonstrated lower mortality in preclinical models of influenza A infection. Even against antigenically drifted strains, it is driven by enhancing phagocytosis and innate cell activation. Critically, TIBT addresses the needs of immunocompromised populations where adaptive immunity often fails. Taking BCG vaccination as a typical example, in order to reduce the risk of infection it cannot only reduce bacterial pneumonia incidence in HIV patients, filling gaps in adaptive immunity with trained innate responses, but also enables monocytes undergo epigenetic changes to secrete inflammatory factors.

Trained immunity can significantly enhance the host's defense against bacterial infections. For example, BCG vaccination primarily used to prevent tuberculosis has been shown to provide heterologous protection against other bacterial infections [197]. The trained immune cells are better able to recognize, phagocytose, and kill bacteria. Higher levels of antimicrobial peptides and proinflammatory cytokines are produced to help to contain the bacterial infection and recruit other immune cells to the site of infection.

In order to limit viral replication and spread, trained immunity can not only play a key role in both the early and late stages of the immune response, but also can produce antiviral cytokines, such as IFN‐γ, more rapidly upon viral exposure [198]. Additionally, trained NK cells and macrophages can target and kill virus, infected cells more efficiently [199]. Some studies have also suggested that certain vaccines may induce trained immunity‐like responses that can enhance the immune response against viral infections, even in the absence of a traditional adaptive immune memory response [200, 201].

Trained immunity is also effective against fungal and parasitic infections. β‐Glucans components of the fungal cell wall can train immune cells to better recognize and respond to fungal pathogens. Trained macrophages and monocytes show enhanced phagocytosis of fungi and increased production of ROS and NO, which are toxic to fungi. In the case of parasitic infections, trained immune cells can produce cytokines that can activate other immune cells involved in the defense against parasites. For example, in helminth infections, trained ILC2s can help to expel the parasites from the body via producing type 2 cytokines.

Unlike traditional vaccines relying on adaptive immunity, TIBVs induce epigenetic and metabolic reprogramming of innate cells through bioactive agents, thereby enabling rapid, broad‐spectrum protection against diverse infections and enhancing immune responses in immunocompromised populations where adaptive immunity often fails [95]. Collectively, TIBT, a revolutionary approach in vaccine development, redefines immune protection by targeting the innate immune system's ability to form long‐term, nonspecific memory, offering a complementary strategy to conventional antigen‐specific vaccines. Certain vaccines not only induce specific immunity but also enhance the function of innate immune cells through trained immunity, providing additional protection against other pathogens, which is particularly significant for individuals with weakened immune systems.

#### 5.1.2 Cancer Immunotherapy

Trained immunity unlocks the antitumor potential of innate cells, providing a complementary approach to adaptive immunity‐based therapies and expanding the scope of cancer immunotherapy [202]. The paradigm shift of trained immunity offers new strategies to augment antitumor immunity, overcome immune suppression in the TME, and improve the efficacy of existing therapies.

Cancer‐related innate cells undergo stable epigenetic changes that upregulate proinflammatory genes and antigen‐presenting molecules. For example, trained macrophages exhibit sustained expression of costimulatory molecules, enhancing their ability to prime tumor‐specific T cells [203]. Meanwhile, cells increase ATP production and biosynthesis of effector molecules killing tumor cells directly through shift from oxidative phosphorylation to aerobic glycolysis. The metabolic switch also supports prolonged cytokine secretion to sustain antitumor responses. In order to trigger signaling cascades that reinforce trained immunity, it is also possible that tumor‐derived antigens or adjuvants activate PRRs on innate cells [204]. For instance, TLR7 agonists induce DC maturation and enhance their ability to cross‐present tumor antigens to T cells.

Trained immunity is being integrated into multiple cancer treatment modalities to improve outcomes. Typically, trained innate cells overcome ICIs resistance by reversing T cell exhaustion and TME immunosuppression [205, 206]. For example, trained DCs increase T cell infiltration into tumors, while trained macrophages polarize from protumor M2 to antitumor M1 phenotypes in the TME. Clinical trials show that TLR7 agonist imiquimod combined with anti‐PD‐1 therapy doubles response rates in metastatic melanoma compared with ICIs alone. Trained immunity is a key target for next‐generation vaccines. DC vaccines loaded with tumor antigens and adjuvants enhance antigen presentation and cytokine secretion via inducing epigenetic reprogramming [207, 208]. mRNA vaccines also activate PRRs via lipid nanoparticles, promoting trained immunity and boosting both innate and adaptive antitumor responses [209]. TME is enriched with immunosuppressive cells, and trained cancer immunotherapies, such as TLR4 agonists or metabolic modulators, reprogram these cells to a proinflammatory state. For example, BCG vaccination reduces recurrence of nonmuscle‐invasive bladder cancer through enhanced macrophage activation and T cell recruitment [206]. While trained immunity holds great promise, several hurdles must be addressed in cancer. Overactivation of trained immunity can exacerbate tumor progression or causing adverse events due to chronic inflammation or autoimmunity. The efficacy of trained immunity strategies varies by tumor type and patient immune profile. Hence, it needs lie on biomarkers to predict response. With long‐term follow‐up required to assess risks, the durability of trained immunity in cancer patients remains unclear.

### 5.2 Role in VITI

VITI, a transformative concept in immunology, is used to describe the ability of vaccines to elicit long‐lasting, nonspecific protection by reprogramming innate immune cells [95]. Unlike adaptive immunity, in order to bridge the gap between immediate and long‐term defense, VITI targeting specific antigens enhances innate cell responsiveness to a broad range of pathogens.

VITI relies on main interlinked processes. On one hand, innate immune cells shift from oxidative phosphorylation to aerobic glycolysis (the Warburg effect), providing energy for enhanced effector function. On the other hand, epigenetic inheritance works. Histone acetylation and DNA methylation stabilize proinflammatory gene expression, creating a memory of prior activation [210]. VITI cannot enhance defense against the vaccine‐targeted pathogen, but also provides broad‐spectrum immunity to unrelated pathogens [211]. For example, BCG vaccination reduces childhood mortality from nontuberculous infections [212]. Based on the benefits, it has profound clinical implications for vaccine design. Targeting metabolic pathways can potentiate VITI, improving efficacy for immunocompromised populations with the elderly and HIV patients. It explains why some vaccines reduce mortality beyond their target pathogen, and offers a strategy to combat emerging threats. Epigenetic inheritance drives long‐term memory, suggesting that VITI could extend vaccine protection without repeated boosters.

Hence, VITI represents a paradigm shift in vaccine development, leveraging innate immunity to enhance both specificity and breadth of protection. As research unravels its molecular basis, VITI will enable next‐generation vaccines that are more effective, durable, and adaptable.

### 5.3 Risk of Overactivation of Trained Immunity

The persistent inflammatory state of trained immunity may disrupt immune tolerance. For example, in individuals with metabolic syndrome or obesity, monocytes are trained to oversecrete proinflammatory factors, and potentially trigger autoimmune diseases due to long‐term exposure to high glucose and lipid environments [213, 214]. In some individuals with allergic constitutions, trained immune cells may trigger severe inflammatory responses and organ damage after be overactivated by COVID‐19 vaccination [215].

In summary, trained immunotherapy as a paradigm shift in immunotherapy harnesses the untapped potential of innate immune memory to expand treatment efficacy and accessibility. As knowledge of its molecular mechanisms deepens, the approach holds the promise to redefine the standard of care for patients with infection, inflammation, and cancer, particularly those who have exhausted conventional options. With exploration from bench to bedside beginning, trained immunotherapy will offer new hope for a disease that has long defied cure.

## 6 Conclusion and Prospects

Trained immunity enables innate immune cells to retain a functional memory of prior stimuli and mount enhanced responses upon secondary exposure [24]. The discovery stemmed from observations that innate immune cells, such as mononuclear macrophages, NK cells, and DCs, undergo persistent molecular and functional reprogramming after encountering stimulus factors. For instance, when monocytes are exposed to β‐glucan, they differentiate into macrophages with epigenetic and metabolic reprogramming. These changes prime macrophages to exhibit enhanced functional activity, such as improved phagocytic capacity, higher ROS/NO production, and increased secretion of proinflammatory cytokines. Other innate cells also display trained immunity. Importantly, these changes are reversible and context‐dependent, allowing innate cells to adapt to varying environmental cues.

The immune system has evolved as a sophisticated defense network that distinguishes self from nonself, eliminates damaging foreign pathogens, and maintains physiological homeostasis. The process operates through three interconnected lines of defense from biochemical or biophysical barriers, innate immunity and adaptive immunity. [216, 217] The essence of trained immunity lies in the adaptive adjustment of innate immunity, where its outcomes depend on a delicate balance between stimulation parameters and host state. This balance gives rise to its “double‐edged sword” attribute. Understanding the duality is critical for translating trained immunity into clinical applications. In order to mitigate harm from epigenetic and metabolic targeting, and vaccine adjuvants, researchers are exploring precision regulation strategies to harness its protective effects.

In summary, research on trained immunity not only deepens knowledge of innate immunity but also holds immense potential to revolutionize immunotherapy. By precisely regulating trained immunity, turning it into a guardian of health rather than a promoter of diseases, novel treatments for infections, inflammation, and cancer will be developed, ultimately improving human health. With the promise of breakthrough solutions for unmet medical needs, the field represents a cutting‐edge frontier in immunology.

## Author Contributions

Conceptualization: M.Y., J.Z., C.Z., and J.G. Writing—original draft: J.Z., C.G., and J.M. Visualization: P.D. and G.L. Writing—review and editing: M.Y., J.G., Z.L., and C.Z. Funding acquisition: J.G. and C.Z. All authors have read and approved the article.

## Funding Information

This work was performed with the support of the Shanghai Frontiers Science Center of Degeneration and Regeneration in Skeletal System (BJ1‐9000‐22‐4002).

## Ethics Statement

The authors have nothing to report.

## Conflicts of Interest

The authors declare no conflicts of interest.

## Data Availability

The authors have nothing to report.
